# Genome-wide in silico identification and expression analysis of beta-galactosidase family members in sweetpotato [*Ipomoea batatas* (L.) Lam]

**DOI:** 10.1186/s12864-021-07436-1

**Published:** 2021-02-27

**Authors:** Fuyun Hou, Taifeng Du, Zhen Qin, Tao Xu, Aixian Li, Shunxu Dong, Daifu Ma, Zongyun Li, Qingmei Wang, Liming Zhang

**Affiliations:** 1 Key laboratory of phylogeny and comparative genomics of the Jiangsu province, School of Life Sciences, Jiangsu Normal University, Xuzhou, 221116 China; 2 Crop research institute, Shandong Academy of Agricultural Sciences/ Scientific Observing and Experimental Station of Tuber and Root Crops in Huang-Huai-Hai Region, Ministry of Agriculture and Rural Affairs, Jinan, 250100 China

**Keywords:** Sweetpotato, β-galactosidase, Gene expression, Stress

## Abstract

**Background:**

Sweetpotato (*Ipomoea batatas* (L.) Lam.) serves as an important food source for human beings. β-galactosidase (bgal) is a glycosyl hydrolase involved in cell wall modification, which plays essential roles in plant development and environmental stress adaptation. However, the function of *bgal* genes in sweetpotato remains unclear.

**Results:**

In this study, 17 β-galactosidase genes (*Ibbgal*) were identified in sweetpotato, which were classified into seven subfamilies using interspecific phylogenetic and comparative analysis. The promoter regions of *Ibbgal*s harbored several stress, hormone and light responsive cis-acting elements. Quantitative real-time PCR results displayed that *Ibbgal* genes had the distinct expression patterns across different tissues and varieties. Moreover, the expression profiles under various hormonal treatments, abiotic and biotic stresses were highly divergent in leaves and root.

**Conclusions:**

Taken together, these findings suggested that *Ibbgals* might play an important role in plant development and stress responses, which provided evidences for further study of bgal function and sweetpotato breeding.

**Supplementary Information:**

The online version contains supplementary material available at 10.1186/s12864-021-07436-1.

## Background

β-galactosidases (EC 3.2.1.23; bgal) widely exist in higher plants. Plant β-galactosidase belongs to the glycoside hydrolase 35 (GH35) families [1], which catalyzes the removal of terminal galactosyl residues from carbohydrates, glycoproteins and galactolipids [2, 3]. In plants, β-galactosidase has been reported to degrade structural polysaccharides in plant cell walls to release free galactose during a variety of biological processes, including cell wall expansion and degradation, metabolic recycling of galactolipids and glycoproteins, and turnover of signaling molecules during ripening [4, 5].

In higher plants, bgals have been grouped into two classes based on their substrate preference [6]. Enzymes in the first class prefer pectic β-(1 → 4)-galactan as the substrate, and enzymes in the other prefer the β-(1 → 3) and (1 → 6)-galactan backbones of arabinogalactan proteins [7, 8]. A typical bgal protein contains the GH35 conserved site in the N-terminal region [9]. Like other glycosidase families, bgal genes are ubiquitously expressed in many plants, such as tomato [2], papaya [10], *Arabidopsis* [11], *Brassica campestris* [12] and rice [13].

Plant *bgal* genes are widely involved in the modification of the architecture of cell walls and intercellular attachments [14, 15]. *bgal* genes also respond to plant growth and development including fruit development and ripening [16, 17], seed germination [18, 19], and root development [20, 21]. In most fruits, *bgal* genes exhibit differential expression patterns during flowering and fruit development [12, 16]. In *Cicer arietinum*, *Canbgal-5* expression is relevant to young and meristematic stages with a high cell division rate, while *CanBGal-1* and *CanBGal-4* are strongly related to later stages of epicotyl growth [3]. In addition, *bgal* genes can be regulated by abiotic and biotic stresses [22]. For example, *Atbgal1* was reported to be induced by salt stress or pathogen attack [23]. Likewise, the transcription level of β-galactosidase in cowpea is reduced under salt treatments [24], and the *bgal* mRNA level in peach is highly suppressed by water stress [25]. In addition, *bgal* genes have been found to play a role in a variety of biological processes through ethylene signal transduction [11, 26]. However, the function of *bgal* has not been studied in sweetpotato (*Ipomoea batatas* (L.) Lam).

Sweetpotato is an important food crop which is widely grown in tropical and subtropical areas, especially in Asia and sub-Saharan Africa. Due to its outcrossing hexaploidy (2n = 6 × =90), the genomic research in sweetpotato is very complicated [27, 28]. So far, no high-quality genome sequence of sweetpotato has been available. Although *bgal* genes are widely isolated from many plant species, its function in sweetpotato remains unknown. In the present study, we firstly identified 17 *bgal* genes (*Ibbgal*) in sweetpotato, and then investigated their phylogeny, motif compositions and predicted cis-elements using various bioinformatics tools. In addition, the expression patterns of these 17 *Ibbgal* gene*s* in different tissues of two cultivars were investigated under three exogenous hormones, two abiotic and one biotic stress conditions. Our study will lay the foundation for further research on the function of *bgal* gene in plants, and provide new insight into different regulatory mechanisms in plant growth through *bgal*-mediated responses to environmental stresses in sweetpotato.

## Results

### Identification and characterization of *Ibbgal* genes in sweetpotato

A total of 17 *Ibbgal* genes were isolated from sweetpotato after local BLAST using the conserved bgal domain. The deduced amino acid sequences of the Ibbgal proteins were used to predict their protein lengths, signal peptides, pI values, molecular weights, sub-cellular localization and the possible N-glycosylation sites (Table 1). Characteristic analysis showed that these 17 Ibbgals were 673 to 1110 aa in length, the predicted MWs and pIs ranged from 74.8 kDa to 125.1 kDa and 5.31 to 6.16, respectively. The predicted localization of most Ibbgals varied and included the chloroplast, vacuole, and nucleus. Only one Ibbgal, Ibbgal7, was found to be located in the extracellular. Signal peptides analysis revealed that all Ibbgals, except for Ibbgal4, Ibbgal5, Ibbgal10, Ibbgal13 and Ibbgal17, contained a signal peptide. The number of N-glycosylation sites varied from 1 to 6, wherein Ibbgal13 and Ibbgal16 contained 6 N-glycosylation sites.

**Table 1:** Gene and protein analysis of bgals in sweetpotato

Gene name	CDS^a^	Length (aa)^b^	MW (kDa)^c^	pI^d^	Subcellular localization	Signal peptides^e^	N-glycosylation site^f^
*Ibbgal1*	2529	842	94.005	5.98	chloroplast	+	3
*Ibbgal2*	2196	731	81.393	8.39	chloroplast	+	2
*Ibbgal3*	2526	841	93.635	7.27	vacuole	+	1
*Ibbgal4*	2529	842	93.578	8.71	vacuole	–	1
*Ibbgal5*	2022	673	74.792	6.32	nucleus	–	1
*Ibbgal6*	2526	841	93.665	7.94	chloroplast	+	1
*Ibbgal7*	2481	826	7.22	9.32	extracellular	+	4
*Ibbgal8*	2541	846	91.829	6.37	vacuole	+	2
*Ibbgal9*	2463	820	92.0858	5.31	vacuole	+	2
*Ibbgal10*	2391	796	89.004	6.83	nucleus	–	4
*Ibbgal11*	2505	834	94.335	8.57	chloroplast	+	5
*Ibbgal12*	2187	728	80.867	9.13	vacuole	+	2
*Ibbgal13*	3333	1110	125.149	5.5	chloroplast	–	6
*Ibbgal14*	2487	828	93.578	8.71	vacuole	+	5
*Ibbgal15*	2475	824	93.72	8.58	chloroplast	+	5
*Ibbgal16*	2412	803	89.731	6.34	chloroplast	+	6
*Ibbgal17*	2145	714	79.382	7.99	chloroplast	–	2

^a^The length of Ibbgals coding sequence

^b^The length of Ibbgals protein

^c^Molecular weight

^d^Theoretical isoelectric point

^e^“+” means contain signal peptide, “–” means lack signal peptide

^f^ Predicted using NetNGlyc1.0

### Conserved motifs and phylogenetic analysis of the Ibbgal proteins

**Fig. 1:**
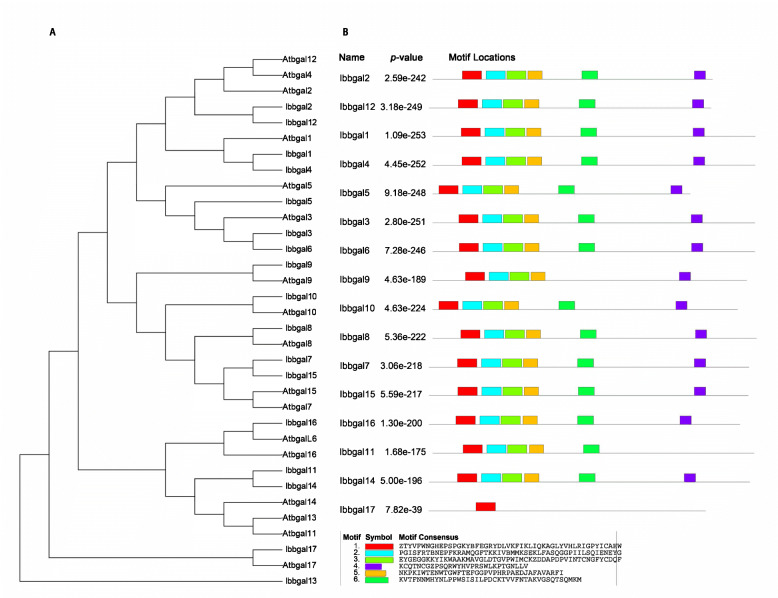
Phylogenetic relationship of Ibbgal proteins and motifs distribution of *Ibbgal* genes. **a.** Phylogenetic relationship among sweetpotato Ibbgals and Atbgals proteins. The uprooted tree was generated using MEGA7.0 by the NJ method. **b.** Motif distribution in *Ibbgal* genes. The motifs were obtained from online tool MEME. The upper part represents the composition and position of motifs of Ibbgals with six motifs shown in distinct colors. The lower part shows the motifs of Ibbgals with the symbol of each residue

**Fig. 2:**
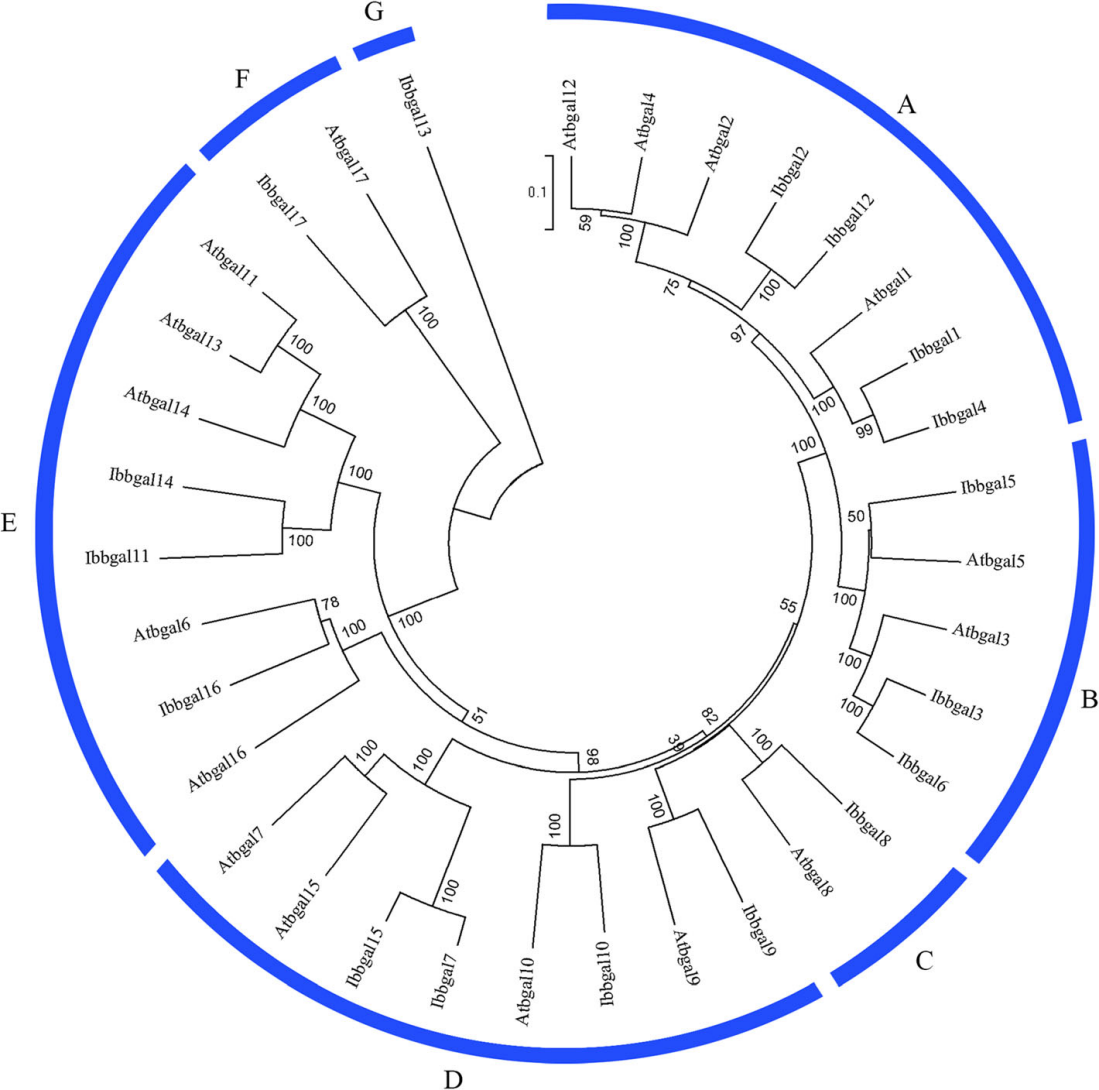
Phylogenetic tree of bgal proteins in sweetpotato, and *Arabidopsis*. The bgal protein sequences of *Arabidopsis* were downloaded from the database of *Arabidopsis* from the NCBI database. The phylogenetic tree was constructed using MEGA 7.0 by the Maximum-Likelihood method analysis with 1000 bootstrap replications. The tree was classified into 7 different subfamilies indicated by outer rings with blue color

In this study, the β-galactosidase active site was found in all Ibbgal proteins. However, all but Ibbgal13 have the active site consensus sequence GGP [LIVM]xQxENE[FY] of the GH35 β-galactosidase family. In addition, all Ibbgal members carried a Gal-lectin domain at the C-terminus of the protein sequence, except for Ibbgal2, Ibbgal5, Ibbgal12, Ibbgal13, and Ibbgal17. Motif analysis showed that motif 1 was found in all Ibbgals except Ibbgal13, and motifs 2–6 were found in all Ibbgals except Ibbgal11 and Ibbgal17 (Fig. 1). A total of 34 *bgal* genes from sweetpotato and *Arabidopsis* were classified into seven subgroups, designated as A, B, C, D, E, F and G using phylogenetic analysis (Fig. 2). Among these groups, groups A and D were the largest groups with four *Ibbgal* genes in each. Groups B and E had three *Ibbgal* genes. However, *Ibbgal9*, *Ibbgal17* and *Ibbgal13* were classified into group C, F and E, respectively.

### Cis-element prediction of *Ibbgal* genes

To understand the potential transcriptional regulatory mechanisms of the *Ibbgal* genes, the cis-elements of each *Ibbgal* promoter sequences were predicted and analyzed (Table 2). The promoters of *Ibbgals* were classified into at least four types of cis-elements, including plant hormone responsive elements, light responsive elements, stress responsive elements, and other elements. Most *Ibbgal* promoters had the GARE (gibberellin-responsive element), ERE (ethylene-responsive element) cis-elements, AuxRE and CATATGGMSAUR motifs which were involved in plant hormone response. Most *Ibbgal* promoters, except *Ibbgal6*, *Ibbgal16* and *Ibbgal17*, contained circadian and EE elements participated in circadian regulation. In addition, at least five light response elements were found in each *Ibbgal* gene, which might be essential for plant growth and development. Interestingly, the *Ibbgal*s contained the MYC-like and ABRE (Abscisic acid response element) cis-elements mediating the response to abotic stresses.

**Table 2:** The putative cis-elements in the promoters of 17 *Ibbgal* genes

Gene	Plant hormone response elements	Stress response elements	Light response elements	Other elements
*Ibbgal1*	ABRE^4^, AuxRE^2^, GARE^2^, TATC-BOX, PYRIMIDINEBOXHVEPB1	box-W^2^, MYC-like^18^, ACGT^10^	INR^8^, GT1-motif^5^, Box 4 ^8^, IBOX^5^, GBOX^3^, GATAbox^10^, GAG-motif, TCT-motif^3^, Box II	EEs, TATA-box^21^, GT^15^, CCAAT-box^3^, AAGAA-motif
*Ibbgal2*	GARE^4^, TGACG-motif2, DPBFCOREDCDC3^2^, CATATGGMSAUR^4^	MBS^2^, MYC-like^18^, ACGT^2^	INR ^3^, IBOX^2^, GATAbox^14^,GAG-motif, TBOX^2^, TCT-motif^2^,AT1-motif	Circadian^2^, TATA-box^18^, CCAAT-box^9^, GCN4-motif, RY-element^4^, GT^12^
*Ibbgal3*	ABRE,ERE, DPBFCOREDCDC3^3^,	MYC-like^16^, ACGT^2^	INR^2^, GT1-motif, IBOX^6^, DRE^2^, GATAbox^15^, GAG-motif, TBOX^3^, TCT-motif, Box II^2^	Circadian, TATA-box^17^, CCAAT-box^6^, RY-element^2^, GT^12^
*Ibbgal4*	ABRE^5^, GARE, AuxRE^2^, PYRIMIDINEBOXHVEPB1	box-W, MYC-like1^8^, ACGT^10^	INR^8^, GT1-motif5, Box 4^8^, IBOX^5^, GATAbox^10^, GAG-motif, TCT-motif^3^, Box II	EEs,TATA-box ^21^,CCAAT-box^ 3^,GT^15^, AAGAA-motif
*Ibbgal5*	ABRE^3^, ERE, GARE, CGTCA-motif^2^, TGACG-motif^4^, DPBFCOREDCDC3^4^, PYRIMIDINEBOXHVEPB1	LRT, box-W, MYC-like1^2^, ACGT^8^, MBS^3^,GT1^8^	INR^6^, GT1-motif^2^, Box 4^3^, IBOX3, GATAbox^15^, Box A, TBOX,TCT-motif^2^, Box II2	Circadian^3^, TATA-box 15, CCAAT-box^6^, Box A,
*Ibbgal6*	ABRE^2^, ERE, GARE^2^, CGTCA-motif^2^, TGACG-motif4, DRE2COREZMRAB^17^, PYRIMIDINEBOXHVEPB1	LRT^3^, MYC-like^10^, ACGT^12^	INR^4^, GT1-motif, Box 4, IBOX^8^, GATAbox^22^, TBOX, TCT-motif^5^, Box II^4^	TATA-box^21^, CCAAT-box^4^, RY-element, GT^13^
*Ibbgal7*	ERE, GARE^2^, AuxRE, CGTCA-motif, TGACG-motif^3^, DPBFCOREDCDC3^2^, CATATGGMSAUR^2^	MYC-like^14^, ACGT^4^, GT-1^5^	INR^4^, Box 4^2^, IBOX^14^, GATAbox^17^	Circadian^4^, TATA-box^17^, CCAAT-box^9^, RY-element^2^
*Ibbgal8*	ABRE^3^, ERE, GARE, DPBFCOREDCDC3^4^, CATATGGMSAUR^4^	LRT^2^, MYC-like^20^, DRE^2^, ACGT^12^, MBS^2^,GT-1^9^	INR^3^, GT1-motif, Box 4^4^, IBOX^8^, GATAbox^18^, TCT-motif^3^, Box II^3^	Circadian^2^, TATA-box^20^, CCAAT-box^3^, RY-element
*Ibbgal9*	ABRE, ERE, GARE^2^	LRT^3^, MYC-like^8^, ACGT^6^, GT-1^5^	INR^3^, GT1-motif, Box 4^2^, IBOX^13^,GATAbox^22^, Tbox^2^, Box II^3^	Circadian^5^, EEs, TATA-box^28^, CCAAT-box^3^,GCN4-motif, RY-element^4^
*Ibbgal10*	ABRE^2^,GARE,DPBFCOREDCDC3, CATATGGMSAUR^2^,PYRIMIDINEBOXHVEPB1	box-W, MYC-like^18^, ACGT^12^, MBS^3^, GT-1^2^	INR^2^, Box 4^3^, IBOX^7^	TATA-box^16^, CCAAT-box^3^, RY-element^3^, Box A^2^
*Ibbgal11*	GARE^3^,CATATGGMSAUR^2^, PYRIMIDINEBOXHVEPB1	MYC-like^8^, ACGT^4^, MBS^2^, GT-1^2^	INR^5^, GT1-motif, Box 4^3^, IBOX^7^, GATAbox^18^, GAG-motif, TBOX^2^, TCT-motif, Box II	Circadian,TATA-box^23^, CCAAT-box^4^,AAGAA-motif, RY-element^2^
*Ibbgal12*	ABRE^3^, ERE, GARE^4^, TGACG-motif, PYRIMIDINEBOXHVEPB1	LRT^3^, box-W, MYC-like^18^, DRE^4^, ACGT^8^,GT-1^8^	INR^8^, GT1-motif, Box 43, IBOX3, GATAbox21, TCT-motif, Box II^2^	Circadian^2^, TATA-box^27^, CCAAT-box^3^,RY-element
*Ibbgal13*	ABRE^3^, ERE, TGACG-motif, DPBFCOREDCDC3	LRT^2^, MYC-like^18^, ACGT^6^, MBS^2^, GT-1^4^	INR^4^, GT1-motif^3^, IBOX^15^, GATAbox^15^, GAG-motif, TBOX, Box II^3^	Circadian, TATA-box^12^, CCAAT-box^4^, RY-element
*Ibbgal14*	ABRE^3^, ERE, GARE, TGACG-motif, DPBFCOREDCDC3^2^, CATATGGMSAUR^4^	LRT^4^, box-W, MYC-like^14^, ACGT^6^, MBS,GT-1^3^	INR^3^, GT1-motif^2^, Box 4, IBOX^10^, GATAbox^18^,CATT, TBOX^3^, Box II^3^	Circadian, TATA-box^13^, CCAAT-box^6^, RY-element^3^
*Ibbgal15*	GARE^2^, DPBFCOREDCDC3^2^	LRT^3^, box-W^2^, MYC-like^28^, GT-1^2^	INR^4^, GT1-motif^2^, IBOX^3^, GATAbox^10^, TBOX^2^, TCT-motif, Box II	Circadian, TATA-box^2^, CCAAT-box^5^, RY-element
*Ibbgal16*	ERE, GARE^2^, DPBFCOREDCDC3^3^, CATATGGMSAUR^2^	LRT^2^, box-W, MYC-like^8^, DRE^3^, GT-1^6^	INR^4^, Box 4^5^, IBOX^2^, GATAbox^13^, GAG-motif, TBOX, TCT-motif	TATA-box^36^, CCAAT-box^3^, RY-element
*Ibbgal17*	ABRE^7^, ERE, GARE^3^, TGACG-motif^4^, DPBFCOREDCDC3^6^, CATATGGMSAUR^2^, GCCCORE	LRT^2^, box-W^3^, MYC-like^10^, ACGT^6^, MBS^2^, GT-1	INR^2^, GT1-motif, Box 4, IBOX^9^, GATAbox^24^, TBOX, Box II	TATA-box^18^, CCAAT-box^4^, GCN4-motif, RY-element^4^

Superscript numbers represent the repeats (2 or more than 2) of each cis-element in the *Ibbgal* promoter, while the others only contain one copy of corresponding cis-element

*ABRE and ACGT* cis-acting elements involved in the abscisic acid responsiveness, *AuxRE* cis-acting regulatory element involved in auxin responsiveness, *AAGAA-motif* cis-element involved in secondary xylem development, *Box A* cis-acting elements of phenylalanine ammonia-lyase, *Box II* part of a light responsive element, *Box-W* fungal elicitor responsive element, *Box 4* part of a conserved DNA module involved in light responsiveness; CATATGGMSAUR, cis-acting element involved in auxin responsiveness, *CCAAT-box* MYBHv1 binding site, *Circadian* cis-acting regulatory element involved in circadian control, *DPBFCOREDCDC3* induced by ABA; DRE, cis-acting element involved in drought response, *EEs* part of evening and circadian response, *ERE* ethylene-responsive element, *GARE* gibberellin-responsive element, *GATA-motif* part of a light responsive element, *Gbox* cis-acting regulatory element involved in light responsiveness, *GATAbox* part of a light responsive element, *GAG-motif* part of a light responsive element, *GCCCORE*, cis-acting element involved in jasmonate responsiveness, *GCN4-motif* cis-regulatory element involved in endosperm, *GT1-motif* light responsive element, *GT-1* cis-acting element involved in the salt stress, *INR* part of a light responsive element, *IBOX* part of a light responsive element, *LTR* cis-acting element involved in low-temperature responsiveness, *MBS* MYB binding site involved in drought-inducibility, *MYC-like*, cis-acting elements of drought-responsive, *PYRIMIDINEBOXHVEPB1* cis- and trans-acting elements involved in gibberellins and abscisic acid responsiveness, *RY-element* cis-acting regulatory element involved in seedspecific regulation, *TATA-box* core promoter element around −30 of transcription start, *TATC-box* cis-acting element involved in gibberellin-responsiveness, *TBOX* part of a light responsive element, *TCT-motif* part of a light responsive element, *TGACG-motif* cis-acting regulatory element involved in the MeJA-responsiveness

### Expression profiles of *Ibbgal* genes in tissues and different root development stages

**Fig. 3:**
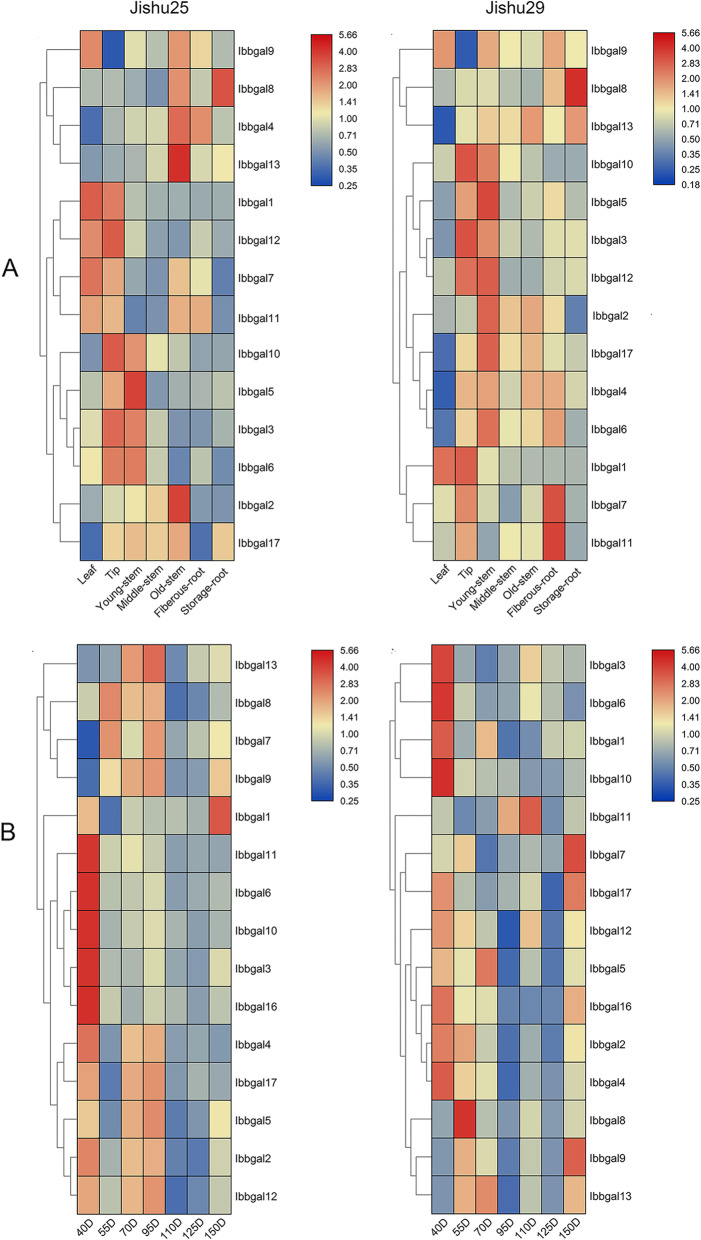
Expression profiles of *Ibbgal* genes in tissues and storage root development of two sweetpotato varieties. **a.** Expression profiles in the tissues. These tissues include the leaf, tip, young-stem, old-stem, fibrous root and storage root. **b.** Expression profiles in the storage root development. Gene expression was detected by qRT-PCR. Log-transformed fold-change data were used for creating the heatmaps by TBtools (v1.059). The coloured scale varying from blue to red indicates relatively low or high expressionv

To identify the potential functions of *Ibbgal* genes, we analyzed the transcript levels of *Ibbgal*s in various tissues of *cv*. Jishu25 and Jishu29, including leaf, stem lip, stem, fibrous root, and storage root. 47% of *Ibbgal*s had similar expression patterns in five tissues of two cultivars (Fig. 3a). For example, *Ibbgal4*, *Ibbgal10*, *Ibbgal13* and *Ibbgal17* were highly expressed in five tissues, whereas *Ibbgal14*, *Ibbgal15* and *Ibbgal16* were poorly expressed in these tissues. Intriguingly, the expression of *Ibbgal4* in fibrous root was significantly higher than that of storage root, while *Ibbgal3* and *Ibbgal10* were expressed at higher levels in lip than other tissues. However, the transcript of *Ibbgal17* mRNA in *cv*. Jishu25 was prominently higher in storage root than fibrous root, whereas that in *cv*. Jishu29 had no significant difference in the roots. Similarly, the expression of *Ibbgal11* had the opposite pattern in the storage and fibrous roots between *cv*. Jishu25 and Jishu29. In root development stages, 6 (35.3%) *Ibbgal *transcripts were down-regulated including* Ibbgal2*, *Ibbgal3*, *Ibbgal4*, *Ibbgal6,* *Ibbgal10*, and* Ibbgal16*, whereas 6 *Ibbgal* transcripts were up-regulated, two *Ibbgal* genes (*Ibbgal14* and* Ibbgal15*) were not detected in root development. It is interesting that the *Ibbgal11* and *Ibbgal12* transcripts had the opposite expression pattern between cv. Jishu25 and Jishu29 (Fig. 3B).

### Expression profiles of *Ibbgal* genes in response to abiotic and biotic stresses

**Fig. 4:**
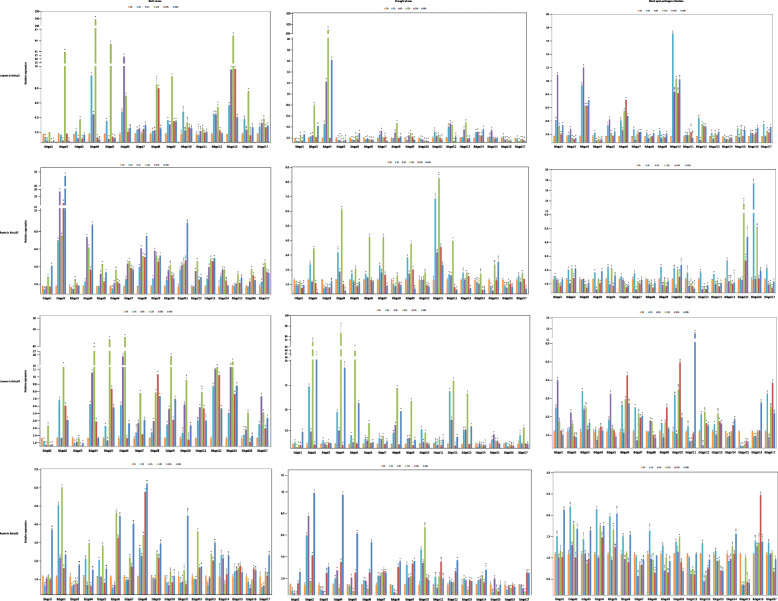
Expression analysis of *Ibbgal* genes under salt, drought stress and balck spot pathogen infection in the leaves and roots of the two cultivars. Gene expression was detected by qRT-PCR. The y-axis represents relative expression, and the data were analyzed using the 2^−△△CT^ method. Bars represent the mean of three biological replicates ± SE. The asteridk indicated that the expression level between the treatment times is significantly different (*P* < 0.05)

Besides their functions in plant growth and development, *Ibbgal* genes may also be involved in response to biotic and abiotic stressses. For sweetpotato, salinity and drought are the most dominant factors which limit the growth and yield among various abiotic stresses. Under salt stress, all *Ibbgal* genes were up-regulated in these two cultivars (Fig. 4). Some genes had the highest expression levels at 12 h in the leaves, whereas other *Ibbgal* genes in roots were expressed at a high level at 6 h and 48 h after salt stress. In addition, *Ibbgal2*, *Ibbgal4*, *Ibbgal5* and *Ibbgal13* in the leaves were up-regulated remarkably by at least 10-fold induction after salt stress. These results indicated that *Ibbgal* genes were involved in salt stress response in sweetpotato. Under drought stress (Fig. 4), all *Ibbgal* genes were up-regulated in the leaves and roots of *cv*. Jishu29, while *Ibbgal3*, *Ibbgal6*, *Ibbgal10*, and *Ibbgal17* were down-regulated in the leaves of Jishu25, *Ibbgal1*, *Ibbgal3* and *Ibbgal16* expression were also reduced in the root of Jishu25. Amongst the up-regulated genes, the expression of *Ibbgal2, Ibbgal4, Ibbgal8, Ibbgal9* and *Ibbgal13* reached the peak at 12 h after stress, and *Ibbgal4* was the most up-regulated gene with at least 81-fold induction in the two cultivars leaves, suggesting that *Ibbgals* in the different cultivars responded to drought treatment differently. Black spot, caused by *Ceratocystis fimbriata*(*C. fimbriata*), is one of the main diseases in sweetpotato production, which seriously affects the quality and yield of sweetpotato. After the pathogen infection, *Ibbgal* genes had different expression patterns in the leaves and roots of these two cultivars (Fig. 4). *Ibbgal5, Ibbgal10*, *Ibbgal11* and *Ibbgal16* transcripts were induced by the pathogen infection in these two cultivars. It is worth noting that *Ibbgal15* expression in the leaves and roots of *cv.* Jishu25 was up-regulated, whereas down-regulated in *cv.* Jishu29. Collectively, these results implied that *Ibbgal* genes in the different cultivars might have different functions under abiotic and biotic stresses.

### Expression profiles of *Ibbgal* genes in response to various hormone treatments

**Fig. 5:**
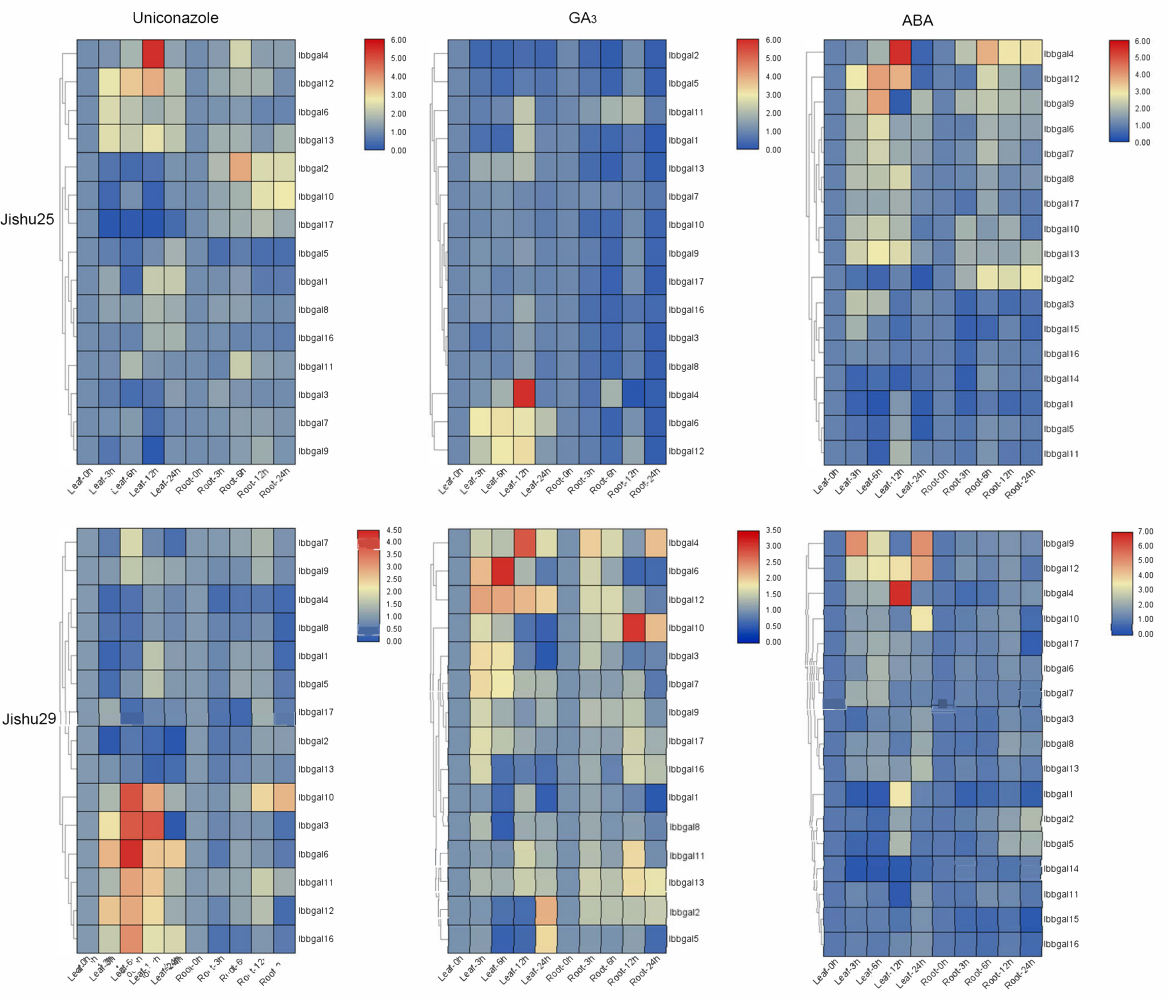
Expression profiles of *Ibbgal* genes after uniconazole, GA_3_ and ABA treatment in the leaves and roots of two cultivars. Gene expression was detected by qRT-PCR. Log-transformed fold-change data were used for creating the heatmaps by Tbtools (v1.059). The coloured scale varies from blue to red, which indicates the low or high expression of each gene

To survey the role of *Ibbgal* genes in plant hormone response, the expression patterns of *Ibbgals* were analyzed under three different hormone treatments. After the uniconazole treatment, the expressions of eight *Ibbgal* genes (including *Ibbgal3*, *Ibbgal6*, *Ibbgal9–12*, *Ibbgal16* and *Ibbgal17*) were induced to the varying degrees in the leaves and roots of these two cultivars (Fig. 5). Interestingly, *Ibbgal4* and *Ibbgal8* expression were up-regulated in *cv.* Jishu25, whereas down-regulated in *cv.* Jishu29 after the uniconazole treatment, indicating that the same *bgal* genes of sweetpotato could respond to uniconazole treatment differently in the different genotypes. After the GA_3_ treatment, the accumulation of four *Ibbgals* (including *Ibbgal4*, *Ibbgal6, Ibbgal11*, and *Ibbgal12*) were unregulated, while *Ibbgal5* was down-regulated in two cultivars (Fig. 5). Among these *Ibbgals*, *Ibbgal4* was the most up-regulated gene, whereas *Ibbgal12* was the least up-regulated gene. In addition, GA_3_ treatment increased the expression of *Ibbgal5* and *Ibbgal10* in *cv.* Jishu29, but decreased the expression in *cv.* Jishu25. For the ABA treatment, most *Ibbgal* transcripts were induced in the leaves of these two cultivars (Fig. 5). In the roots, most *Ibbgal* transcripts were up-regulated under the stress, except for *Ibbgal1* and *Ibbgal15*. Among the up-regulated genes, *Ibbgal4* was significantly induced in *cv*. Jishu25, while it was slightly up-regulated in *cv*. Jishu29. These data indicated that sweetpotato *bgal* genes might play pivotal roles in hormone-response pathways.

## Discussion

β-galactosidase participates in cell wall biogenesis and modification during plant growth [15, 17]. In this study, 17 β-galactosidase cDNAs were isolated from sweetpotato, which have the same number of β-galactosidases as in *Arabidopsis*, tomato and peach [17, 29]. All Ibbgals except Ibbgal13 had the active site consensus sequences GGP[LIVM]xQxENE[FY]. Most Ibbgal members contained a Gal-lectin domain at the C-terminus, which might be responsible for substrate specificity of bgals [11, 29]. In addition, most Ibbgals were predicted to have signal peptides in the N-terminus, which might be involved in cell wall-related biological processes [29]. The phylogenetic tree was constructed using the bgal proteins from sweetpotato and *Arabidopsis*, which was similar to those of tomato and rice [13, 29]. This result implied that the bgals in the same branch might have similar and distinct functions, and bgal diversification might occur in the early stage of plant evolution. *Ibbgal4* and *Atbgal1* of groups A shared the same clade, suggesting that they might have similar functions.

In a previous study, Esteban et al. (2005) found that *bgal* genes participate in the development of vegetative organs in *Cicer arietinum* [3]. *Atbgal* genes were reported to have differential tissue-specific expression patterns [11]. Similarly, the expression patterns of *Ibbgal*s were distinct in different tissues of sweetpotato in this study. Most *Ibbgal* genes were expressed in all tissues, whereas *Ibbgal14*, *Ibbgal15* and *Ibbgal16* had low expression levels in five tissues. The results are consistent with the observations in *Arabidopsis* reported by Gantulga et al. (2009) [30]. A number of cis-elements related to development, such as GCN4_motif, TATA box and RY-element, were found in the promoter of *Ibbgal* genes [31, 32], suggesting that these genes might be related to the development of sweetpotato. *Ibbgal2–4*, *Ibbgal6*, *Ibbgal10*, *Ibbgal12* and *Ibbgal17* were highly expressed in the early stage of root development. Previous reports have shown that *Atbgal5* is involved in root elongation through modifying the cell wall [21, 33]. Lovas et al. (2003) found that *Stubgal83* might participate in root and tuber development by altering the metabolic sugar status of the leaves [34]. Thus, we deduced that *Ibbgal*s might be associated with root development by modifying the cell wall and carbohydrate metabolism. Further study is needed to investigate the function of *Ibbgal* genes during root development in sweetpotato.

To date, increasing evidences manifest that *bgal* genes are involved in response to various hormone, biotic and abiotic stresses. *PaGAL3* and *PaGAL4* trancripts in avocado fruit were found to be inhibited by ethylene and ripening signals [26]. In plant coleoptile tissues, auxin-induced increase of elongation rate is closely associated with the β-galactosidase activity [3, 35]. Li et al. (2003) reported that the β-galactosidase genes in calamander were down-regulated through IAA, JA and ethylene after infection by fungus *C. acutatum* of citrus flower [36]. Our study showed that the upstream region of all *Ibbgal*s contained three to seven cis-elements related to phytohormone responses, such as GARE, ERE, AuxRE, CATATGGMSAUR. GARE and PYRIMIDINEBOXHVEPB1, which are involved in plant hormone responses [37, 38]. In this study, the expression of eight *Ibbgal* genes was significantly up-regulated by the uniconazole treatment. Meanwhile, the majority of the *Ibbgal* genes were regulated by the GA_3_ treatment in leaves and stems of these two cultivars. ABA is a requisite factor in response to stress, senescence, and fruit development [39, 40]. We found that most *Ibbgal* genes were induced under ABA treatment. These results revealed that *Ibbgal* genes mignt play important roles in phytohormone responses. Spadoni et al. (2014) found that the expression levels of *bgal* genes decreased in peach fruit after hot water treatment [25]. Several *bgal* genes are regulated by abiotic and biotic stresses in *A. thaliana* and *Brassica campestris* [12, 23, 41]. In addition, the cis-elements related to stress responses, such as MYC-like, LRT, W-BOX, MBS and ACGT-motif, have been found in the promoter region of *Ibbgal* genes, which might regulate gene expression during biotic and abiotic stresses [42, 43]. Similarly, our result showed that most *Ibbgal* transcripts were related to salt stress, drought stress, ABA treatment and pathogen infection. For example, the expression of all *Ibbgal4* was greatly up-regulated by salt and ABA treatments in the leaves of sweetpotato. Taken together, these *Ibbgal* genes play essential functions in response to biotic and abiotic stresses and their related signal transduction pathways.

In particular, *Ibbgal*s exhibited different stress and hormone response patterns between leaves and roots, and have distinct expression profiles in the two cultivars. There are different in root pectin content from sweetpotato cultivars. β-galactosidase functions in the degradation of galactan side chains of pectin leading to cell wall loosening and softening [44, 45], suggesting that β-galactosidase may be involved in the regulation of the pectin content, and different bgal-mediated pathways might be activated in the storage root development. In respond to stresses, the accumulated sugar has been reported to involve in osmotic adjustments to sustain cell structure and photosynthesis in plant [46, 47]. Pandy et al. (2017) found that loss of sugar was the key regulator for activation of the cell wall hydrolase during senescence [48]. β-galactosidase under abiotic and biotic stresses might be induce the initial structural modification of cell wall and activated to degrade cell wall polysaccharides for producing sugar. Therefore, *Ibbgal* genes were mainly up-regulated expressed under abiotic and biotic stresses. Further studies need to be performed to investigate the functions of bgals on the stress-response system in sweetpotato.

## Conclusion

We characterized 17 *Ibbgal* genes and then analyzed their motif compositions and N-glycosylation site. Based on the phylogenetic analysis, the bgals were divided into seven subgroups. We also investigated their promoter regions and sub-cellular location. In addition, we systematically investigated the expression profiles in different tissues, and different development stages of storage roots, as well as the expression of the bgals under six different environmental treatments. The diversification of the bgal genes provides a solid foundation for further elaborating the bgal-mediated stress-response system in sweetpotato.

## Methods

### Identification and isolation of *Ibbgal* genes in sweetpotato

To identify *Ibbgal* genes, we performed local BLAST and domain search for genes containing the conserved domain of bgals in two transcriptase databases (SRP068179 and CRA000288). The obtained transcript sequences were translated and analyzed by the PFAM program (http://pfam.xfam.org) to examine the presence of the bgal conserved domains. The transcripts encoding proteins which were less than 120 amino acids were removed. The bgal domain was confirmed by analyzing transcripts deduced proteins screened in the NCBI BLAST. If two or more transcripts had the identity of amino acids equal to or higher than 97%, only one of these transcripts was kept in the final list of the genes. Pooled samples including 9 tissues of shoot, leaf, stem, fibrous root, storage toot, flower, salt-treated, drought-treated and ABA-treated plants were collected from two sweetpotato cultivars (Jishu25 and Jishu29). The total RNA was isolated from the pooled sample using TRIzol, and cDNA was synthesized using a reverse transcription Kit (Transgene, China). To isolate the *Ibbgal* genes, the gene-specific primers were designed used for PCR amplification (Additional file 1). The obtained sequences were compared to the corresponding transcripts, and the related protein data are summarized in Table 1.

### Protein properties, N-glycosylation site and subcellular location of the *Ibbgal* proteins

The molecular weights (MW) and isoelectric points (pI) of *Ibbgal* genes were analyzed using the ExPasy server (http://web.expasy.org/protparam/) [49]. N-glycosylation site analysis of *Ibbgal* genes was conducted using the NetNGlyc 1.0 server (http://www.cbs.dtu.dk/services/NetNGlyc/) [12]. The WoLF PSORT tools (https://wolfpsort.hgc.jp/)were used to predict the subcellular location of the Ibbgal proteins [50].

### Conserved motifs, phylogenetic analysis and promoter region prediction of the Ibbgal proteins

The conserved domains were identified by the online program SMART (http://smart.embl-heidelberg.de/). These 17 Ibbgal protein sequences were aligned with the MEME server (http://meme-suite.org/tools/meme). The protein sequences of Ibbgals were aligned, and the phylogenetic tree was constructed using the Neighbor-Joining (NJ) method of MEGA software 7.0 [51]. The bgal protein sequences from different species, including *Arabidopsis* [29], were obtained based on the description in the literature or downloaded from the Plantgdb database (http://www.plantgdb.org/). The promoter sequences (1.5 kb) of *Ibbgal* genes was obtained from sweetpotato genomic DNA (https://ipomoea-genome.org/#), and then the cis-acting elements were predicted using the PLACE tool (http://www.dna.affrc.go.jp/PLACE/) [52].

### Quantitative real-time PCR analysis

To investigate the function of 17 *Ibbgal*s in sweetpotato, the expression patterns were analyzed in various organs, hormone treatments, abiotic and biotic stresses using qRT-PCR. The primer sequences of the examined genes were listed in Table S2 (Additional file 2). Total RNA was extracted from the frozen samples by using an RNAprep pure plant kit (TIANGEN, Beijing, China) according to the manufacturer’s instructions. qRT-PCR was performed using a Roche LightCycler® 480II system under the following conditions: 95 °C for 15 s, followed by 40 cycles of 95 °C for 15 s, 55 °C for 15 s and 72 °C for 15 s. The *Ib-Actin* gene was used as an internal reference to evaluate the relative gene expression level. The experiments were conducted for three replicates, and the data were calculated according to the 2^−△△CT^ method [53].

### Plant materials and stress treatments

The seedlings of two sweetpotato cultivars (*cv*. Jishu25 and Jishu29) were collected from the Crop Ressearch Institue, Shandong Academy of Agricultural Sciences, China. The uniform seedlings of the two cultivars were grown in the Hoagland solution at 26 °C under a photoperiod of 16 h light/8 h dark. When the seedlings had five to six functional leaves and adventitious roots of 8 to 10 cm, these seedlings were subjected to six different stresses, respectively. To study the expression patterns under these stresses, the adventitious roots of seedlings were submerged in the solution containing 150 mM NaCl, 20% PEG 6000, 100 mM ABA, 50 mg/L uniconazole, and 50 mg/L gibberellic acid (GA_3_) respectively [54]. For black spot pathogen treatment, *C. fimbriata* conidia was collected after growing in potato dextrose agar (PDA) at 28 °C for 7 days, then were diluted to 1 × 10^4^ spores/mL with sterile water, and then the roots of sweetpotato seedlings were cultivated in the 1 × 10^4^ spores/mL conidia suspension. The treated roots and leaves were collected after 0, 3, 6, 12, 24, and 48 h. To investigate the *Ibbgals* transcript levels in different tissues, the fifth expanded leaves, lips, stems, fibrous roots and storage roots of the two cultivars were sampled at 125 days after transplanting, and the storage roots were sampled at 40, 55, 70, 95, 110, 125 and 150 days after transplanting in the sweetpotato field.

### Statistical analysis

Statistical analysis was performed using the SPSS software package (v13.0), and the datas were presented as means of three replicates. Differences between means were subjected to ANOVA, and the statistical significance of the difference between means was calculated with Duncan’s new multiple ranges test and marked with asterisks at *p* < 0.05.

## Supplementary Information

**Additional file 1: Table S1** The gene-specific primers of *Ibbgal* genes.

**Additional file 2: Table S2** The primer sequences of 17 *Ibbgal* genes for qRT-PCR analysis

## Data Availability

The datasets analyzed during the current study are available in the GenBank and TAIR repository, the accession numbers of 17 *Ibbgal* genes are MW566714- MW566730, and the bgal protein sequences of *Arabidopsis* were downloaded from the TAIR database (http://www.arabidopsis.org/).

## Abbreviations

ABA: Abscisic acid
BLAST: Basic local alignment search tool
bgal: β-galactosidase
GH35: Glycoside hydrolase 35
GA_3_: Gibberellins
IAA: Indolyl-3-acetic acid
JA: Jasmonic acid
MW: Molecular weights
NJ: Neighbor joining
pI: Isoelectric points
qRT-PCR: Quantitative reverse transcription polymerase chain reaction
