# Supplementary material for: Genome-wide in silico identification and expression analysis of beta-galactosidase family members in sweetpotato [Ipomoea batatas (L.) Lam]
Source: BMC Genomics. 2021 Feb 27;22:140. doi: 10.1186/s12864-021-07436-1 (PMC7912918; doi:10.1186/s12864-021-07436-1)
Supplement: Supplementary file 1 — Additional file 1: Table S1 The gene-specific primers of Ibbgal genes. [file 12864_2021_7436_MOESM1_ESM.docx]

Table S1 The gene-specific primers of *Ibbgal* genes.

| Gene name | Primer |
| --- | --- |
| *Ibbgal1* | S: 5-ATAGTGCAGAAGAATAAGGGTC-3  A: 5-CAATCGAGGTGGGAACAA-3 |
| *Ibbgal2* | S:5-GTGTTGGTGGGTGGTGGG-3  A:5-GGTGAGTGTCCCTTTCCTA-3 |
| *Ibbgal3* | S:5-GAGGGAGAAATTGAGAGGTGGAGCC-3  A:5-TCAATGCCATTGGGAAGGGGTGC-3 |
| *Ibbgal 4* | S:5-GCCCAGAAAGGCTGAAGA-3  A:5-CCTTTAGCCTATGATGTTGTG-3 |
| *Ibbgal 5* | S:5-TTTGCTGTGCTTGGTGGTG-3  A:5-TGCCACCTTATCATAACCTTGA-3 |
| *Ibbgal 6* | S:5-TCTTGAGGATTATAGTTTGAGA-3  A:5-GTATCACCACTCGTTAATCTAA-3 |
| *Ibbgal7* | S:5-ATGGCATCTTTTCTCACTAG-3  A:5-TAATCTAATCAAAGCCAACA-3 |
| *Ibbgal8* | S: 5-GGGCCTTTGATGAGGGTG-3  A:5-TTAGGCACAGGAAGCTTCTAC-3 |
| *Ibbgal9* | S:5-AGAATGGCACCGAGAACTT-3  A:5-CCCAGCTCCTAGTCACTCA-3 |
| *Ibbgal10* | S:5-GCACTCCATCTACCGCTCTT-3  A:5-TGTAACTATGGCTTGGACAAT-3 |
| *Ibbgal11* | S:5-ATGGGTAGGGCAAGTTGTGT-3  A:5-TATCTAGTCGTCTTTGCCCT-3 |
| *Ibbgal12* | S:5-GAATAGTAAGAAAACGAGAAGAA-3  A:5-AGCCTCCCCTAAAATCAA-3 |
| *Ibbgal13* | S:5-CCTGGAAAGGGGATGGCT-3  A:5-TTCAGAGTCTAGGCTAAACAAG-3 |
| *Ibbgal14* | S:5-ACATGGCTCAACCACGTG-3  A:5-GGTCACAAATAACGGCAAAA-3 |
| *Ibbgal15* | S:5-ATGGCATCTTTACTCACTAG-3  A:5-CTAACAAGTAGCTTCAATGG-3 |
| *Ibbgal16* | S:5-GATTGTTAGAACTTGATGCC-3  A:5-CAGATGTCATTGCGAAGAA-3 |
| *Ibbgal17* | S:5-TGGAGCAGAAATGGAGTT-3  A:5-AACCTGGATAGTAAGTTTGTAG-3 |
