# Supplementary material for: Genome-wide in silico identification and expression analysis of beta-galactosidase family members in sweetpotato [Ipomoea batatas (L.) Lam]
Source: BMC Genomics. 2021 Feb 27;22:140. doi: 10.1186/s12864-021-07436-1 (PMC7912918; doi:10.1186/s12864-021-07436-1)
Supplement: Supplementary file 2 — Additional file 2: Table S2 The primer sequences of 17 Ibbgal genes for qRT-PCR analysis [file 12864_2021_7436_MOESM2_ESM.docx]

Table S2 The primer sequences of 17 *Ibbgals* genes for qRT-PCR analysis

| Gene name | Primer |
| --- | --- |
| *Ibbgal1* | S: 5-GGAGCAGATAAATACCACCAG-3  A: 5-TCCATAGGCACTTCCGACT-3 |
| *Ibbgal2* | S:5-TTCACTACCCAAGAAGCACTC-3  A:5-ATTCCATCCCAGGCACAT-3 |
| *Ibbgal3* | S:5-TGGCTATGAAAGGGTTTACTG-3  A:5-CTGGCTTGTATGGTTTATTGG-3 |
| *Ibbgal 4* | S:5-AGGGAGGTATGATTTAGTGAAGT-3  A:5-GAAGCTGACACCCGGAAC-3 |
| *Ibbgal 5* | S:5-GGCATTGGACATGGGTAG-3  A:5-AGGATCGAGGAACGTGGT-3 |
| *Ibbgal 6* | S:5-AAAAGCAGCAACCTCTAACA-3  A:5-TCCAGCATAATGGCAACC-3 |
| *Ibbgal7* | S:5-TTGATGACCGCAATAAGTT-3  A:5-CCATTCACCCAAGCAACT-3 |
| *Ibbgal8* | S: 5-GACTATGGACCTCGTGCTAAG-3  A:5-ACAGGACCACCAAATGAAAT-3 |
| *Ibbgal9* | S:5-TTGAGGAAACCGATAAAGC-3  A:5-AGAAATCACGGAGGAGGAA-3 |
| *Ibbgal10* | S:5-AGGTTGGGTTGAAAGGAGA-3  A:5-GCACTTGTTAGGGCTGAAT-3 |
| *Ibbgal11* | S:5-GTGGATCAACGGGAAAGG-3  A:5-GATCGTCGCTCACAGACTTG-3 |
| *Ibbgal12* | S:5-GGGCAGATGGCTGTAGGT-3  A:5-CCAGTCCAGAGTTCGGTAAA-3 |
| *Ibbgal13* | S:5-ATGCTGGACCGTGCTGTA-3  A:5- CACCACCACCTTCATAGTGTA -3 |
| *Ibbgal14* | S:5- TCCCGAATAGCGGAGCCT -3  A:5- CCGAAAGACCTACCGAATGG -3 |
| *Ibbgal15* | S:5- TCGGGTGGACCATACATT -3  A:5- CTCATACACGGTTGAAGACA -3 |
| *Ibbgal16* | S:5- TTCCGATCTGATAACGAACC -3  A:5- GCTGCCCAACGAACATAA -3 |
| *Ibbgal17* | S:5- GACAGGGCACAGGTCTTT -3  A:5- CTCCCAACATTCTCCACTTA -3 |
| *Ib-Actin* | S:5- CTGGTGTTATGGTTGGGATGG-3  A:5- GGGGTGCCTCGGTAAGAAG-3 |
